# Quantification of Warfarin in Dried Rat Plasma Spots by High-Performance Liquid Chromatography with Tandem Mass Spectrometry

**DOI:** 10.1155/2016/6053295

**Published:** 2016-12-12

**Authors:** Alexander Chernonosov

**Affiliations:** Institute of Chemical Biology and Fundamental Medicine, Siberian Branch of Russian Academy of Sciences, Academician Lavrentiev Avenue 8, Novosibirsk 630090, Russia

## Abstract

This paper presents the development and validation of a novel method for quantification of the oral anticoagulant drug warfarin in dried plasma spots (DPS) by high-performance liquid chromatography with tandem mass spectrometry (HPLC-MS/MS). Blood plasma was chosen as a biological fluid to preclude the influence of the hematocrit on the results of the analysis. A 30 *μ*L sample of rat plasma was placed onto Whatman 903 Protein Saver Card and was allowed to dry. A single DPS is sufficient for preparing eight 3.2 mm discs, each containing approximately 1.5–1.6 *μ*L of plasma. Warfarin extraction from one 3.2 mm disc was carried out by adding 200 *μ*L of the acetonitrile : water mixture (1 : 1, v/v) containing 10 mM NH_4_COOH (pH 4.0), with incubation on a shaker at 1000 rpm for 1 h at 25°C. After chromatographic separation, warfarin and coumachlor (an internal standard) were measured using negative-ion multiple-reaction monitoring with ion transitions *m*/*z* 307 → 161 for warfarin and *m*/*z* 341 → 161 for the internal standard. The working range of this method is 10–10,000 ng/mL. Within this range, intra- and interday variability of precision and accuracy was <13% and recovery was 82–99%. The results indicate that the new method requires only small plasma samples and may be useful for pharmacokinetic research on warfarin.

## 1. Introduction

Warfarin is an oral anticoagulant widely used for the treatment of arterial and venous thromboembolism as well as for primary and secondary prevention of these disorders [1]. The most common indications for warfarin therapy are arterial fibrillation, venous and arterial thrombosis, systemic embolism, and mechanical heart valves. Warfarin therapy is lengthy, sometimes life-long. Careful monitoring of coagulation by measuring the international normalized ratio of prothrombin time (INR) is necessary to personalize the treatment. The INR should be in the range of 2 to 3. At INR < 2, warfarin therapy is ineffective, but INR > 4 may cause bleeding. Accordingly, the research interest in warfarin is still substantial: interactions with other drugs are actively studied [2, 3], and new formulations and modifications of the drug are being developed [4, 5]. Initially, the analytical method of high-performance liquid chromatography (HPLC) with ultraviolet (UV) spectrum detection was used for the quantification of warfarin [6–8]. In these works, warfarin was isolated from plasma or serum by solid-phase extraction on a semipermeable surface guard column [6] or by liquid/liquid extraction with ice-cold acetonitrile [7] or with diethyl ether [8]. Although a 100 *μ*L [6, 7] or 1 mL aliquot of plasma [8] was used, such an analytical method was questionable due to low specificity and sensitivity of UV detection. Therefore, several methods of HPLC with mass spectrometry (MS) and HPLC with tandem mass spectrometry (MS/MS) have been developed for warfarin quantification in single-reaction monitoring mode [5, 9, 10] or multiple-reaction monitoring mode [4, 11–13]. Warfarin has been isolated by solid-phase extraction [9, 12], liquid/liquid extraction [4, 5, 10, 13], or simple protein precipitation with ZnSO_4_ [11]. Nevertheless, the volume of plasma required for one analysis has been rather high: 50 *μ*L [10], 200 *μ*L [4, 12, 13], 300 *μ*L [9], or even 1 mL [4]. At the same time, in such studies, blood and plasma samples are usually collected from small animals like mice and rats. This situation imposes restrictions on the volume of the analyzed sample of whole blood, plasma, or serum. A possible solution is to use dried blood spots (DBS) or dried plasma spots (DPS) on filter paper, as first suggested by Dr. Robert Guthrie in the 1960s for the detection of phenylalanine for diagnosis of phenylketonuria in neonates [14]. A single DPS corresponding to 30 *μ*L of plasma is sufficient for preparing eight 3.2 mm discs, each containing approximately 1.5–1.6 *μ*L of plasma. The samples collected in this manner are convenient for transport and storage, while stability of the samples is improved. Application of MS allows DPS and DPS to be increasingly used in clinical trials for the analysis of various small-molecule drugs [15–23]. The aim of the present study was to develop and validate a rapid and robust method of HPLC-MS/MS for simultaneous quantification of warfarin in DPS with a simple extraction procedure.

## 2. Experimental

### 2.1. Reagents

Warfarin (C_19_H_16_O_4_), coumachlor (chlorowarfarin, C_19_H_15_ClO_4_, used as an internal standard), formic acid, and ammonia were purchased from Sigma-Aldrich (St. Louis, MO, USA). Formic acid and ammonia were used to prepare formate buffer (pH 4.0, 0.1 M).

Acetonitrile, ethanol (80%), and methanol of MS grade were purchased from Panreac AppliChem (Barcelona, Spain). Water was purified by means of a Milli-Q system from Millipore Corp. (Bedford, USA). Nitrogen gas (ultrapure, >99.9%) was produced by an Agilent 5183-2003 nitrogen generator (Agilent Technologies, USA).

### 2.2. Equipment and HPLC-MS/MS Conditions

Mass spectrometry analysis was carried out in the Core Facility of Mass Spectrometric Analysis (ICBFM SB RAS). An Agilent 1200 Series HPLC system (Agilent Technologies, USA) consisted of a micro degasser (G1379B), bin pump (G1312A), autosampler (G1367B), thermostatted column compartment (G1316A), and diode array detectors (G1315B). The system was controlled by a software package for data processing (MassHunter, v.1.3; Agilent Technologies, USA).

Chromatographic separation of the samples was conducted on an EcoNova ProntoSil-120-3-C18 (2 × 75 mm, 3 *μ*m) analytical column (EcoNova, Russia), with a Zorbax Eclipse XBD-C18 guard column (4.6 × 12.5 mm, 5 *μ*m). The flow rate was 200 *μ*L/min for separation and up to 400 *μ*L/min for washing, and the gradient was composed of solvent A (0.01% formic acid in water), and solvent B (99% acetonitrile in aqueous 0.01% formic acid). The chromatography program consisted of 100% solvent A for 0–0.5 min, followed by 0% A and 100% B from 0.5 to 3.0 min, followed by washing (0% A and 100% B) from 3.0 to 4.0 min (flow rate 200 to 400 *μ*L/min), and finally 100% A and 0% B from 4.0 to 5.0 min (flow rate 400 to 300 *μ*L/min). The total run time was 5 min. The autosampler and the column temperature were held at 25°C.

MS/MS detection was performed on an Agilent 6410 QQQ mass spectrometer (Agilent Technologies, USA) equipped with electrospray ionization (ESI) source. Analytes were detected in negative ionization mode using multiple-reaction monitoring. The capillary voltage was set to 4000 V, and the gas temperature was set to 300°C. The nebulizer gas pressure and flow were 30 psi and 8 L/min, respectively.

Dwell time was set to 80 ms, and full width at the half-maximum was 0.7* m*/*z* for quadrupoles one (Q1) and three (Q3). The ion transitions were* m*/*z* 307 → 161 for warfarin (collision energy 10 V, fragmentor voltage 120 V) and* m*/*z* 341 → 161 for the internal standard coumachlor (collision energy 10 V, fragmentor voltage 120 V). Signal output was captured and processed with the MassHunter software v.1.3.

### 2.3. Preparation of Calibration Standards and Quality Control Samples

Warfarin and the internal standard were dissolved in acetonitrile to prepare a 10 mg/mL stock solution. The warfarin stock solution was diluted with acetonitrile to prepare intermediate stock solutions that were added to blank rat plasma to create calibration standards with warfarin concentrations of 10, 25, 100, 250, 500, 1000, 2500, 5000, and 10,000 ng/mL. Three quality control samples (low-concentration quality control sample [LQC], medium-concentration quality control sample [MQC], and high-concentration quality control sample [HQC]) were prepared in the same way by spiking blank rat plasma with a corresponding stock solution to attain final warfarin concentrations of 50, 800, and 4000 ng/mL. All stock solutions, standards, and quality control samples were freshly made on the day of the analysis and were stored at 4°C before use. The calibration standards and quality control samples (each consisting of 30 *μ*L of rat plasma) were placed on a Whatman 903 Protein Saver Card (GE Healthcare, USA) to fill the circles on the card and were air dried completely for 12 h. After that, 3.2 mm circles of DPS were cut out by means of a DBS Puncher, and each circle was placed in a 1.5 mL Eppendorf tube.

### 2.4. Sample Preparation

An extraction solution containing 2.5 ng/mL internal standard was prepared in the acetonitrile : water mixture (1 : 1, v/v) containing 10 mM NH_4_COOH (pH 4.0). Sample extraction was carried out by adding 200 *μ*L of the extraction solution, with incubation on a shaker (TS-100C; BioSan, Latvia) at 1000 rpm for 1 h at 25°C. After centrifugation for 10 s at 1000 ×g, 170 *μ*L of the solution was transferred to a 300 *μ*L vial, and a 50 *μ*L aliquot was injected onto the LC-MS system.

### 2.5. Assay Validation

#### 2.5.1. Calibration and Linearity

Calibration curves were constructed using nine concentrations of warfarin. Each calibration standard was analyzed in duplicate on 3 days, except for the lower limit of quantification (LLOQ), which was analyzed in triplicate. For each curve, the absolute peak area ratios of warfarin to the internal standard were calculated and plotted against the nominal analyte concentration. The calibration curves (warfarin peak area divided by internal-standard peak area on the *y*-axis and warfarin concentration on the *x*-axis) were built on the basis of the least square linear regression fit (*y* = *ax* + *b*) with the weighting factor of 1/*y*.

#### 2.5.2. Accuracy and Precision

These parameters were assessed by analysis of six replicate quality control samples on 2 days, followed by analysis of 12 replicate quality control samples on the third day, for a total of 24 samples at each quality control level (LQC, MQC, and HQC). Intraday accuracy and precision were determined by means of the 12 replicates on day 3, and interday accuracy and precision were calculated using all 24 quality control samples. The ratio (%) of the calculated mean concentration to the nominal concentration was defined as accuracy (% bias). Relative standard deviation (% RSD) was calculated from the quality control values and was used to estimate the precision.

#### 2.5.3. Extraction Recovery and Matrix Effects (Ion Suppression)

These parameters were evaluated for LQC, MQC, and HQC in quadruplicate. For extraction recovery analysis, peak areas corresponding to extracts of the fully cut out 5 *μ*L plasma spots of quality control samples were compared with the peak areas resulting from direct injection of the standards (without extraction) at the same nominal concentrations after reconstitution in the working solution of the internal standard.

For evaluation of MS ion suppression, extracts of warfarin-free fully cut out 5 *μ*L plasma spots were spiked with LQC, MQC, and HQC samples of warfarin. The peak areas corresponding to quality control samples added to plasma extracts were compared to standards without extraction at the same nominal concentrations after reconstitution in the working solution of the internal standard. All experiments were conducted at the three quality control levels with four replicates [24].

#### 2.5.4. Stability

Stability was evaluated at LQC and HQC levels in triplicate. Bench top stability was tested by analyzing DPS that were left out on the bench top in the postal envelope at room temperature for 7, 14, or 21 days before analysis. Refrigeration stability was determined for DPS that were placed into a refrigerator (4°C) in a postal envelope, with incubation for 7, 14, or 21 days before analysis. Freeze-thaw stability was assessed for samples that were subjected to three freeze-thaw cycles at 24-hour intervals from −80°C to room temperature prior to analysis. The results on all the tested samples were compared with the recovery from samples that were freshly prepared (100% control).

## 3. Results and Discussion

To enable preclinical studies on small samples of plasma, a robust and simple HPLC-MS/MS method for warfarin quantification in DPS was developed and validated here. The LLOQ showed the ability of the method to work with miniscule samples: a 3.2 mm disk of a DPS contains approximately 1.5–1.6 *μ*L of blood plasma. The use of DPS simplifies warfarin extraction from the matrix containing a lot of proteins, without centrifugation before HPLC-MS/MS analysis. The method was implemented in the same way as described in a study on chemical reduction of warfarin in vitro [25] and was validated according to the US Food and Drug Administration guidelines for validation of bioanalytical methods [26].

### 3.1. Extraction Optimization

To maximize recovery of warfarin from DPS, several solvents were tested as extraction reagents: methanol, methanol : water (1 : 1, v/v), water, ethanol, acetonitrile, acetonitrile : methanol (1 : 1, v/v), acetonitrile : water (1 : 1, v/v), and acetonitrile : water (1 : 1, v/v) containing 10 mM NH_4_COOH (pH 4.0). The last solvent yielded the best extraction. Besides, when this solvent was used as the extraction reagent, the peaks during chromatographic separation were sharp and contributed to a higher signal-to-noise ratio.

### 3.2. Chromatographic Separation

**Figure 1:**
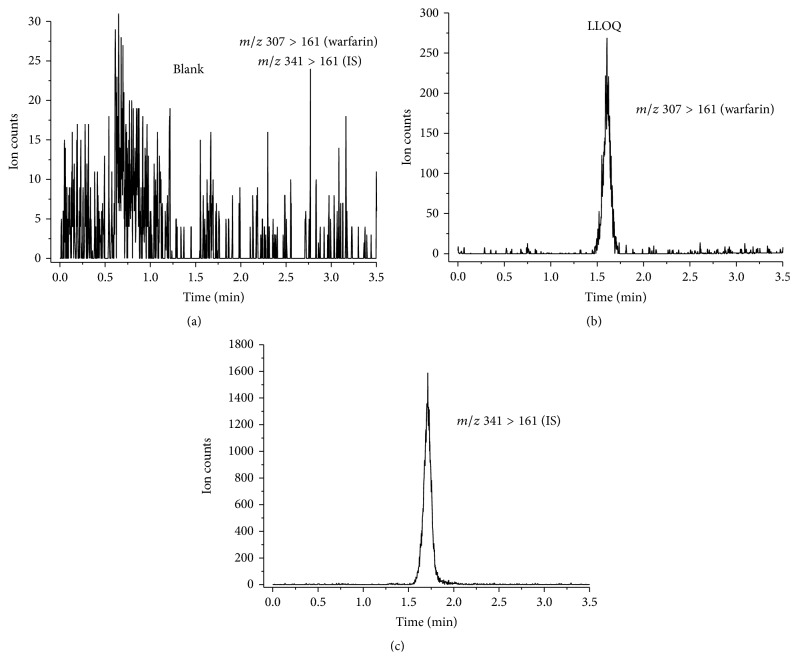
Representative multiple-reaction monitoring chromatograms of blank plasma (a) and blank plasma spiked with 10.0 ng/mL warfarin (b) or 2.5 ng/mL internal standard (c).

A representative chromatogram of warfarin at the LLOQ is shown in Figure 1. Warfarin and the internal standard were identified by retention time and ion transitions of the analytes. The retention time was approximately 1.6 and 1.71 min for warfarin and the internal standard, respectively. Because warfarin and chlorowarfarin have different molecular masses and are detected by MS/MS, good chromatographic separation is not required. The longer chromatogram with the well-separated warfarin and internal standard did not show a change in the total ion count and ion suppression or enhancement.

### 3.3. Assay Validation

#### 3.3.1. Calibration and Linearity

Calibration curves were obtained across the concentration range 10–10,000 ng/mL for warfarin, with a correlation coefficient (*r* ^2^) greater than 0.994 for all curves. Samples at the LLOQ revealed acceptable accuracy and precision (RSD and bias of LLOQ were within ±18% and ±10%, resp., Table 1), and the signal-to-noise ratio was above 10 : 1 (Figure 1).

#### 3.3.2. Accuracy and Precision

The intraday and interday accuracy (% bias) and precision (% RSD) were determined at warfarin quality control concentrations of 50, 800, and 4000 ng/mL. Assay bias ranged from −1.6% to 12.8%, while RSD was from 7.2% to 11.8% (Table 1). In all cases, bias and RSD values were within ±13% for all quality control samples.

#### 3.3.3. Recovery, Process Efficiency, and the Matrix Effect

The extraction recovery and process efficiency toward warfarin ranged from 82.2% to 99.5% and from 83.9% to 105.4%, respectively. Overall, the recovery and process efficiency were consistent and reproducible. The deviation of measured concentrations by 1.9–8.8% from neat samples indicates that the matrix effect was negligible (Table 2).

#### 3.3.4. Stability

Bench top, refrigeration, and freeze-thaw stability were assessed for warfarin at the LQC and HQC levels. The samples were found to be stable for at least 2 weeks at room temperature (bench top) and in a refrigerator (at 4°C). This time should be sufficient for sample transportation (if necessary, by regular mail). Additionally, three freeze-thaw cycles had no significant effect on the stability of warfarin in DPS. The mean measured concentrations of warfarin during the 2 weeks ranged from 90% to 121% of freshly analyzed samples, indicating adequate stability under all the conditions tested. After the third week, the samples showed signs of degradation (down to 85% of the original level, Table 3).

### 3.4. Possible Practical Application

The proposed method was developed for clinical and preclinical studies of pharmacological effects of warfarin. The linearity range of the method is 10–10,000 ng/mL, which is suitable for studies on biological samples from humans and animals, where the working range of warfarin is 10–800 [27] and 100–8000 ng/mL [4, 5, 28], respectively. Sometimes in studies on samples from humans, the LLOQ of less than 10 ng/mL is required; this parameter may be achieved by minor modifications of the proposed method, for example, by cutting bigger discs of DPS.

## 4. Conclusions

The method presented here offers a validated quantitative analysis of warfarin in DPS samples. The assay was validated in terms of sensitivity, specificity, linearity, accuracy, and precision as well as stability at room and refrigeration temperatures. This method seems to be suitable for clinical or preclinical pharmacokinetic studies of warfarin when small plasma samples are required.

## Figures and Tables

**Table 1:** Intra- and interday accuracy (% bias) and precision (% RSD) for LLOQ and quality control samples of warfarin.

Level	Nominal conc. (ng/mL)	Intraday^a^		Interday^b^	
		% bias	% RSD	% bias	% RSD
LLOQ	10	1.9	12.5	10.0	18.0
LQC	50	11.9	7.9	6.5	11.8
MQC	800	−1.6	7.2	0.8	9.7
HQC	4000	12.8	11.6	6.7	10.8

^a^Three replicates for LLOQ; 12 replicates for LQC, MQC, and HQC each.

^b^Nine replicates for LLOQ; 24 replicates for LQC, MQC, and HQC each.

**Table 2:** Extraction recovery, process efficiency, and the matrix effect.^a^

Nominal conc. (ng/mL)	Extraction recovery (%, mean)	Process efficiency (%, mean)	Matrix effect (%, mean)
50	96.9	105.4	108.8
800	99.5	101.4	101.9
4000	82.2	83.9	102.1

__^a^ *n* = 4 for each QC level.

**Table 3:** Stability for quality controls (LQC and HQC) of warfarin and its alcohol metabolites.^a^

Time	Target (ng/mL)	Stability					
		Bench top		Refrigerator		Three freeze-thaw cycles	
		% of target	% RSD	% of target	% RSD	% of target	% RSD
24 h	50	—	—	—	—	127.9	11.9
	4000	—	—	—	—	120.9	1.4
1 week	50	104.1	6.8	121.9	4.4	—	—
	4000	101.0	4.2	94.6	3.6	—	—
2 weeks	50	119.8	20.0	121.2	5.2	—	—
	4000	90.1	3.5	90.1	2.1	—	—
3 weeks	50	86.8	13.1	105.6	7.9	—	—
	4000	99.3	3.6	85.0	4.5	—	—

^a^Data are presented as means (*n* = 3) at LQC and HQC levels.
